# Effect of non-sentinel metastasis on adjuvant treatment decisions and survival in Z0011 eligible non-screened detected breast cancer population

**DOI:** 10.3332/ecancer.2021.1324

**Published:** 2021-11-26

**Authors:** Sanjit Kumar Agrawal, Vishal Kewlani, Noopur Priya, Abhishek Sharma, Joydeep Ghosh, Sanjoy Chatterjee, Rosina Ahmed

**Affiliations:** 1 Department of Breast Oncosurgery, Tata Medical Center, 14, MAR(E-W), DH Block(Newtown), Action Area I, Newtown, Kolkata, West Bengal 700160, India; 2 Department of Medical Oncology, Tata Medical Center, 14, MAR(E-W), DH Block (Newtown), Action Area I, Newtown, Kolkata, West Bengal 700160, Kolkata, India; 3 Department of Clinical Oncology, Tata Medical Center, 14, MAR(E-W), DH Block(Newtown), Action Area I, Newtown, Kolkata, West Bengal 700160, Kolkata, India

**Keywords:** breast cancer, SLNB, Z0011, India

## Abstract

**Background:**

The Z0011 trial results have shown that axillary lymph node dissection (ALND) can be avoided in cT1-2 patients undergoing breast conservation surgery with 1–2 metastatic sentinel lymph nodes (SLNs). We compared the clinicopathological characteristics of the Z0011 eligible non-screen detected breast cancer patients’ cohort with the Z0011 trial study population. Additionally, we have explored the effect of non-sentinel metastasis on adjuvant treatment decisions and survival.

**Methods:**

The details of early breast cancer (EBC) patients fulfilling Z0011 eligibility criteria were retrieved from a prospectively maintained database (2013–2017) and electronic medical records. We used Statistical Package for the Social Sciences 25 and Stata V15 for the data analysis.

**Results:**

128/194 (66%) sentinel lymph node biopsy positive patients had fulfilled the Z0011 inclusion criteria. Compared to the Z0011 study, our cohort patients were younger, with more aggressive disease (higher T2, Grade 3), had a higher rate of macrometastasis (82.8% versus 58.8%) and non-SLN metastasis (48% versus 27%). The information gained by ALND had changed decisions for chemotherapy in 3% and no change of radiotherapy in Z0011 eligible patients. Further nodal positivity in completion ALND was not significantly associated with overall survival (*p* = 0.86) and disease-free survival (*p* = 0.5).

**Conclusion:**

Z0011 eligible Indian EBC patients are significantly different from the Z0011 study population, with younger age of presentation, higher grade, a higher rate of both SLN macro metastasis and non-SLN positivity. The impact of non-sentinel metastasis on adjuvant treatment decisions and survival is minimal.

## Introduction

In the last three decades, surgical management of axilla has evolved from axillary lymph node dissection (ALND) to sentinel lymph node biopsy (SLNB) in the majority of breast cancer patients [1–4]. SLNB alone reduces the morbidity of ALND procedure like shoulder stiffness, pain and paresthesia in the arm and risk of lymphedema [1]. Before 2010, ALND was the standard procedure for patients with metastatic sentinel lymph nodes (SLN). However, in the last decade, three randomised controlled trials (Z0011, International Breast Cancer Study Group (IBCSG) – 01 and After Mapping of the Axilla: Radiotherapy Or Surgery (AMAROS)) have published their long-term results, with non-inferior survival outcomes following omission of ALND in a selected group of early breast cancer (EBC) patients with metastatic SLNs [5–7].

The landmark prospective randomised controlled trial (RCT) Z0011 included EBC patients undergoing breast conservation surgery (BCS) with tumour size <5 cm and up to two metastatic SLN receiving planned radiotherapy and systemic treatment. This study has randomised patients in two arms – ALND in the standard arm and no further axillary treatment in the experimental arm [8]. The authors published initial results in 2011 with 6.3 years of follow up showing equivalent survival in both arms [9]. After this publication, there was extensive debate among breast cancer experts worldwide about the applicability of the trial results in clinical practice. The study was extensively criticised, with incomplete accrual, unclear radiation fields, mainly consisting of good biology tumour and many more issues [10, 11]. Despite the criticism, the study’s early results have led to significant changes in practice and guidelines, and many surgeons now omit ALND in SLN positive patients, who fulfil the trial inclusion criteria [12]. The authors published long term 10 years survival outcomes in 2018, and reported no difference between the two arms in overall survival (OS) (83.6% versus 86.3%, non-inferiority *p* = 0.02), Disease-free survival (DFS) (78.2% versus 80.2%, *p* = 0.32) and locoregional relapse-free survival (81.2% versus 83%, *p* = 0.41) [6]. The Z0011 authors reported that the ALND arm had more seroma (*p* < 0.001), paraesthesia (*p* < 0.001), wound complications (*p* = 0.0016) and lymphedema rate (*p* < 0.001) compared to the SLNB arm [13].

Several validation studies have supported the results of the Z0011 trial, mainly in Western populations with a high proportion of screen-detected breast cancer [12, 14, 15]. In the United Kingdom, the ongoing RCT POSNOC (Positive Sentinel Node – Adjuvant therapy alone versus Adjuvant therapy plus Clearance or Axillary Radiotherapy) is recruiting patients undergoing either BCS or mastectomy with up to two SLN macrometastases, who are randomised to either no further axillary treatment or additional treatment (ALND or axillary radiotherapy) [16]. In Asia, data regarding the application of Z0011 results in clinical practice is minimal [17]. Apart from trial critics, oncologists in Asia are hesitant to de-escalate axillary surgery in patients with metastatic SLNs, with concerns about the comparatively advanced stage of presentation in the non-screened population [18]. In a survey of axillary management practice amongst surgeons of India, only 15.8% of the respondents have reported omission of ALND in Z0011 trial eligible patients [19]. In contrast, many oncologists from the developed countries have accepted the Z0011 results and are moving further by extrapolating Z0011 results in mastectomy patients with 1–2 metastatic SLNs [20, 21].

The objectives of the present study were to compare our population of non-screened breast cancer with the Z0011 patients in terms of demographics, histopathological characteristics and survival. In addition, we analysed the impact of additional information gained from completion ALND on decisions related to adjuvant treatment and survival.

## Methodology

The retrospective study was done with approval from the Institutional Review Board (EC/WV/TMC/013/19). All clinically node-negative EBC patients who underwent SLNB from 2013 to 2017 were included. We excluded patients who had prior breast surgery, pure ductal carcinoma *in situ*, and SLN positive with isolated tumour cells only. Data were extracted from the prospectively maintained REDcap (Vanderbilt University, Nashville, TN) database and hospital management system. Patients follow-up data were retrieved until 30 April 2020 to identify events of interest (death and recurrence).

The SLNB was performed by using either radioisotope, methylene blue (MB) or both as per the availability of the radioisotope. Microfiltered (0.22 micron) 99 m Tc-S was injected at least 2 hours before surgery in the peritumoral region. 2 ml of 1% MB dye was injected in the periareolar area just before the surgery, followed by 5 minutes of whole breast massage. A Gama camera (Europrobe3, EURORAD S.A2) was used to identify the radioactive nodes. Lymph nodes with radioactive count >10% of the tumour count were considered as hot nodes. All hot, visibly blue and clinically suspicious nodes were labelled as SLN and send for frozen section, which was reported as per AJCC and ASCO guidelines [22, 23]. All patients with positive SLNs on the frozen section or subsequent paraffin section had completion ALND.

**Figure 1.**
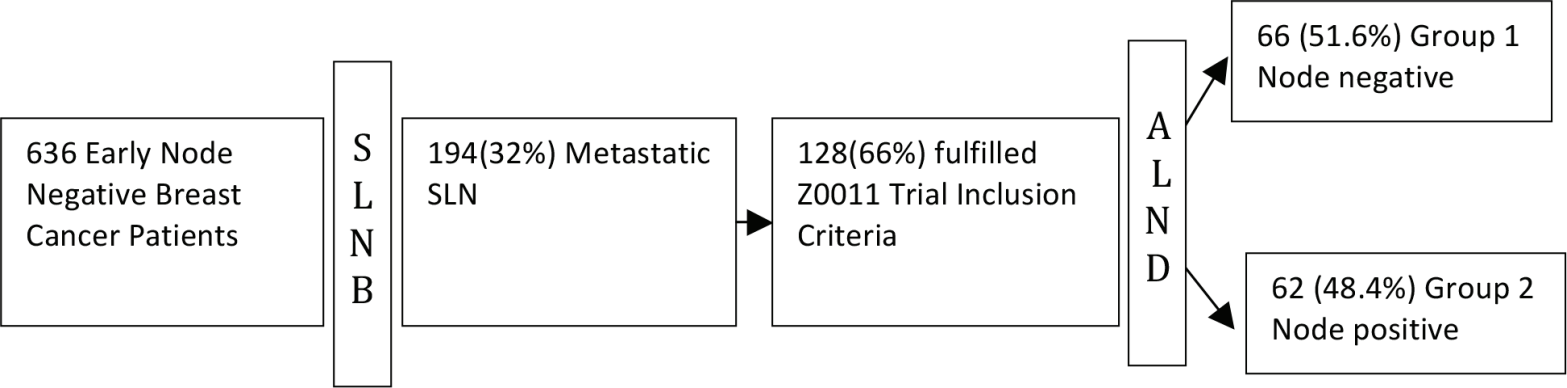
Patient selection (study schema).

All patients with metastatic SLNs were screened for the inclusion criteria of the Z0011 study. Patients who have fulfilled the Z0011 inclusion criteria were included in the analysis. Clinical and histopathological characters of the present cohort were compared with the Z0011 study population. All Z0011 eligible patients were divided into two groups on the basis of further metastatic nodes being identified on completion ALND – group one included patients who had no further metastatic nodes, and group two included patients with metastatic nodes (Figure 1). The possibility of extrapolation of Z0011 results to mastectomy patients was explored by including mastectomy patients with relevant tumour and SLN characteristics, with Z0011 eligible patients (Z0011 + mastectomy) as a separate analysis. The effect of further information gained by completion ALND on the stage of cancer and adjuvant treatment decisions was assessed by comparison between the two groups as defined above. The additional benefit of ALND on survival was evaluated by a comparative analysis of DFS and OS between two groups.

All patients received adjuvant treatment as per multidisciplinary tumour board recommendations. Predict NHS tool was used for prognostication, and chemotherapy was recommended to all patients with survival benefit ≥4% at 10 years [24]. Radiotherapy was recommended for all patients who had BCS. Post mastectomy radio therapy (PMRT) was prescribed to patients with a Cambridge index score ≥ 3 [25]. Predict NHS tool and Cambridge index were used for decision making due to the non-availability of national cancer guidelines during time of the study. The aforementioned validated tools are easy to use in resource constraint settings. All patients were treated using linear accelerators, and treatment planning was done using CT scan based 3d-conformal techniques and details of the treatment planning procedure is available from our previous publication [26]. Tumour bed boost was given post BCS, and 2 cm bolus (1 cm above and below the mastectomy scar, of 5 mm thickness) was used in post-mastectomy patients. The axilla was not irradiated as per institutional protocol, and all patients have received breast, chest wall, and supraclavicular radiotherapy. We used a field-based technique for coverage of the chest wall and supraclavicular area. The lateral edge of the tangential field was placed such that the apex of the axilla was spared. Matched supraclavicular field radiotherapy using Case bow’s technique [27] was used such that there was no overlap between supraclavicular fossa and tangential field.

Endocrine therapy was recommended to all patients with positive oestrogen receptor (Allred score ≥ 3) or positive progesterone receptor (Allred score ≥ 3). Trastuzumab was recommended to all HER 2 positive patients.

Summary statistics were represented by a percentage, median and interquartile range. Univariate analysis was done by Chi-square/Fisher exact test, and multivariate analysis was done with binary logistic regression. DFS was defined as the duration from the date of diagnosis to the date of recurrence (locoregional or distant), and OS as the duration from the time of diagnosis to death from any cause. Survival analysis was done by the Kaplan–Meier method, and log-rank *p*-value was used to compare the two groups. Patients who have either completed follow up without event or lost to follow up were censored. All *p* values were two-tailed, and *p* < 0.05 was considered to be statistically significant. Statistical Package for the Social Sciences version 25 (IBM Corporation, Chicago, IL) and STATA version 15 (College Station, TX: Stata Corp LLC) were used for analysis.

## Results

636 patients had SLNB during the study period, and SLNs were identified in 606 patients, with an identification rate of 95.2%. 194/606 (32%) patients had metastatic SLN. 128/194 (66%) patients fulfilled the inclusion criteria of the Z0011 study (Figure 1). The patients were divided into two groups as per metastatic lymph nodes in completion ALND – 66 (51.6%) had no further positive nodes (Group 1), and 62 (48.4%) had additional metastatic nodes (Group 2)**.**

The median age was 51 years (IQR 44, 59), and the median tumour size was 2.5 cm (IQR 2.2, 3.2). Table 1 compares the demographic, clinical and histopathological characteristics of our Z0011 eligible cohort with participants from the original study. We found significant differences, with our group being younger and with more aggressive disease (higher proportions of invasive ductal cancer, T2, grade 3 and progesterone receptor negativity). The SLNs had macro metastasis in 82.8% of patients versus 58.8% in the Z0011 study. Following completion of ALND, further metastatic nodes were identified in 48%, compared to 27% in the ALND arm of Z0011. In Table 2, we have summarised factors associated with further nodal metastasis in the completion ALND. The tumour stage (T2 > T1) and the SLN macro metastasis were significantly associated with further nodal metastases.

Table 3 summarises the impact of further nodal positivity by completion ALND on tumour stage and adjuvant treatment decision compared to SLNB only. Although the tumour and nodal stage were upgraded in 19.5% of our Z0011 eligible cohort, multidisciplinary team (MDT) recommendations for chemotherapy were changed in only 3% of patients. In the Z0011 + mastectomy cohort, the tumour and nodal stage were upgraded in 18.7%, with chemotherapy recommendations being changed in 2.2%. PMRT decisions did not change for any patients. Only 5/48 (10%) of mastectomy patients with low-risk features (T1N1, G1-2) were recommended no radiotherapy as per the Cambridge Index score.

As per our records, in Z0011 eligible patients, 96% and 94% of patients have received recommended chemotherapy and radiotherapy, respectively. The HER 2 neu receptor status was known for 124/128 Z0011 eligible patients. 22/124 (17.7%) were HER 2 receptor-positive, and only 8/22 (36%) have received trastuzumab due to financial constraints. The compliance to endocrine therapy was good in our cohort, and >95% of the patients have followed prescribed hormonal treatment at the time of data collection.

**Figure 2.**
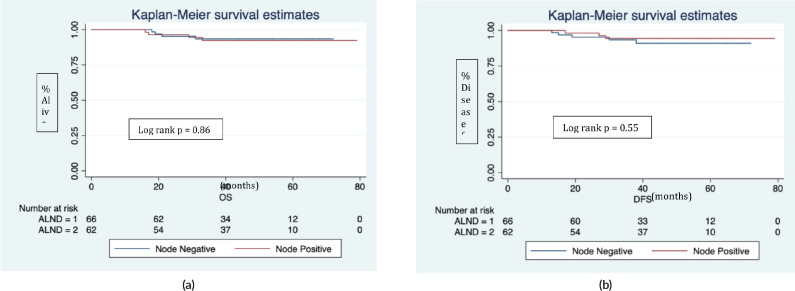
Overall and disease-free survival in Z0011 eligible patients. (ALND = 1) represents node negative and (ALND = 2) node positive in completion ALND. In a median follow up of 40 (IQR 29, 54) months. (a): Four deaths in each ALND group (1&2). (b): Five recurrences in ALND (node negative) and three in ALND (node positive).

The median follows up was 40 (IQR 29, 54) months. In the Z0011 eligible cohort (*n* = 128), there were eight recurrences (six distant, two locoregional) and eight deaths (five disease progression, one accidental, one leukaemia and one myocardial infarction). The 5 years estimated OS was 92.8%, and DFS was 92.6%. Further nodal positivity in completion ALND was not significantly associated with OS (*p* = 0.86, Figure 2a) and DFS (*p* = 0.5, Figure 2b).

**Figure 3.**
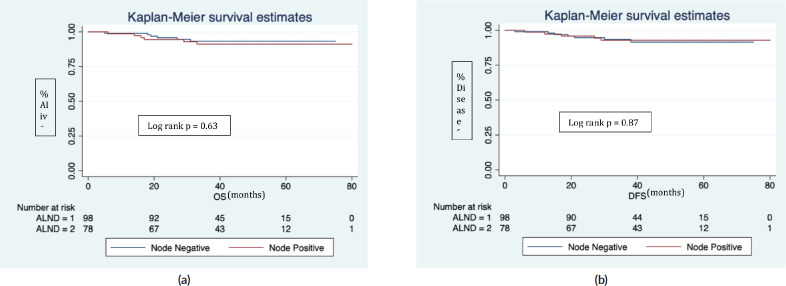
Overall and disease-free survival in Z0011 eligible patients + mastectomy. (ALND = 1) represents node negative and (ALND = 2) node positive in completion ALND. In a median follow up of 40 (IQR 29, 54) months: (a): Six deaths in each ALND group (1&2). (b): Seven recurrence in ALND (node negative) and five in ALND (node positive).

In Z0011 eligible + mastectomy cohort (*n* = 176), there were 12 recurrences (10 distant, 2 locoregional) and 12 deaths (9 disease progression, 1 accidental, 1 leukaemia and 1 myocardial infarction). The 5 years estimated OS and DFS were 93.1%. Further nodal positivity in completion ALND was not significantly associated with OS (*p* = 0.63, Figure 3a) and DFS (*p* = 0.87, Figure 3b).

## Discussion

In India at present, the omission of ALND in Z0011 eligible patients, in general, is not a routine practice [18], so it is difficult to make a direct assessment of the impact of ALND following positive SLNB. To compare the outcome of these two approaches, we chose a group of patients who would fit the Z0011 study inclusion criteria, but all of whom had ALND. We then divided the study cohort into two groups depending upon further nodal positivity in the histopathology report of ALND (Figure 1). The comparative analysis between two groups (node-negative versus node-positive in completion ALND) enabled us to estimate the impact of non-sentinel nodal metastasis on adjuvant treatment and survival.

Compared to Z0011 study participants, EBC patients in our cohort had significantly larger tumours, more aggressive biology, higher SLN macrometastasis rate and more nodal metastasis in completion ALND. The difference in tumour characteristics may be due to the lack of a breast cancer screening program in India, where patients generally seek physician’s advice for breast cancer symptoms like lump or nipple discharge [28]. Additionally, breast cancer in India presents more commonly in young women with aggressive biology such as G3 and triple negative [29]. The higher stage of presentation and aggressive biology are the primary concerns of the oncologist in India for omitting ALND in Z0011 eligible patients. Besides, there is a fear that omission of ALND may leave residual pathological nodes, which in turn may lead to poorer survival [18]. A National Cancer Data Base study published similar concerns in experts from the western region and have concluded that younger patients and those with HER2 positive and triple-negative subtype were more likely to undergo ALND even though fulfilling eligibility criteria’s of the Z0011 study [30]. However, another recently published study has highlighted overtreatment for EBC patients in India, resulting in patients facing unnecessary financial burdens, treatment-related complications and reduced quality of life [31].

In our study, SLNs were the only metastatic node in 51.6% of patients. T2 stage and macro metastasis in SLN were factors significantly associated with the finding of further metastatic nodes in completion ALND. The aggressive tumour characters like high grade, ER negativity and younger age were not significantly associated with the residual axillary burden. Similar results of lack of association of aggressive tumour biology and non-SLN positivity in Z0011 eligible patients was reported in multiple studies [32, 33]. The non-association of aggressive tumour characteristics with residual axillary burden supports omission of completion ALND in patients fulfilling Z0011 eligibility criteria.

Systemic therapy in EBC patients is now, in general, decided by tumour biology and genomic predictors rather than regional nodal status [34, 35]. In combination with clinical and histopathological characteristics by using predict NHS tool [24], 89% of our patients were advised adjuvant chemotherapy based on information derived from the SLNB result alone. With additional information available from ALND, a further four patients (3% overall) were also recommended chemotherapy. Thus, after the MDT recommendation, 86% of our patients had chemotherapy, which is substantially higher than the Z0011 study population, in which 58% had received chemotherapy in SLNB and 57.9% in ALND arm [8].

Several studies have examined whether it is possible to extrapolate the results of Z0011 to mastectomy patients. However, some authors have raised concerns that information of additional positive nodes in ALND may play a crucial role in decision making for radiotherapy [36]. In our Z0011 + mastectomy cohort, we found that the additional information from completion ALND did not change the radiotherapy recommendation for any patient. All Z0011 + mastectomy patients in our cohort were mainly T1-2, N1 breast cancer patients and the role of PMRT improving survival in this group is supported by level one evidence like EBCTCG metanalysis [37].

The 5-year estimated OS in the present cohort was 92.6%, which is similar to the Z0011 study, which reported a 5-year OS rate of 92.5% for SLNB group and 91.8% in ALND group [8]. In spite of the relatively advanced stage of presentation, in our cohort, the 5-year estimated DFS was 92.6% which is better than the Z0011 study, which reported 5-year DFS of 83.9% in the SLNB group and 82.2% in the ALND group. The lower rate of distant recurrences may be attributed to the higher proportions of patients receiving chemotherapy in our cohort (86% versus 58%) [8]. Additionally, in our cohort, 83% of patients received endocrine therapy compared to 46% in the Z0011 study population [8]. When we compared survival between additional node-negative and node-positive patients following completion ALND, there was no significant difference in OS and DFS. The absence of survival differences between the two groups may indicate that it may be appropriate to avoid ALND in these patients, and systemic therapies may be adequate to take care of additional nodal metastases.

The approach to patients with positive SLNs is gradually changing. The St. Gallen consensus (2019) panel has recommended avoiding ALND if regional nodal irradiation is planned for post-mastectomy patients who otherwise fulfil Z0011 eligibility criteria [34]. Another comparative analysis of mastectomy and BCS patients who had metastatic SLNs without completion ALND showed equivalent survival results, irrespective of the type of surgery, with 4-year DFS and OS of 94.8% and 97.8% in the mastectomy group versus 90.1% and 92.6% in the BCS group [38]. In our study too, the 5-year estimated OS and DFS were almost the same in Z0011 + mastectomy patients compared to Z0011 eligible BCS patients. These results suggest that it may be possible to extrapolate Z0011 results to mastectomy patients with tumours <5 cm and 1–2 metastatic SLNs, to avoid the morbidity associated with ALND.

Our study has some potential limitations. It is a retrospective data analysis, which may have some selection biases. At the time of analysis, the follow-up time was 40 (IQR 29, 54) months with a low event rate, which may contribute to no significant difference in survival between the two groups. Longer-term follows up with more events may adequately power the study to see any difference in survival between two groups. The majority of HER 2 receptor-positive patients in our cohort have not received trastuzumab, which may affect the survival analysis. The chemotherapy decisions were based on clinical risk predictors based on NHS predict score, and it may change by using genomic scores in the current practice.

## Conclusion

In conclusion, Z0011 eligible Indian EBC patients are significantly different compared to the Z0011 study population, with younger age of presentation, higher grade, a higher rate of both SLN macro metastasis and non-SLN positivity. Despite these aggressive characters, the incremental impact of completion ALND on adjuvant chemotherapy and radiotherapy decisions change was minimal. The non-sentinel metastasis was not associated with poor survival in present cohort. These results may give food for thought to LMIC oncologists to avoid completion ALND in EBC patients (BCS with *T* < 5 cm, 1–2 positive SLN).

## Conflicts of interest

The authors declare no conflicts of interest.

## Funding

There was no funding received for this study.

## List of abbreviations

ALND, Axillary lymph node dissection; DFS, Disease-free survival; EBC, Early breast cancer; NCDB, National Cancer Data Base; OS, Overall survival; PMRT, Post mastectomy radio therapy; RCT, Randomised controlled trial; SLN, Sentinel lymph node; SLNB, Sentinel lymph node biopsy.

## Authors’ contributions

**Conceptualisation**: Sanjit Kumar Agrawal; **methodology**: Sanjit Kumar Agrawal; **data collection**: Vishal Kewlani, Noopur Priya, Sanjit Kumar Agrawal, Rosina Ahmed; **data analysis**: Vishal Kewlani, Sanjit Kumar Agrawal; **writing – original draft preparation**: Sanjit Kumar Agrawal; **writing – review and editing**: all authors; **final approval of manuscript**: all authors.

## Figures and Tables

**Table 1.** Clinicopathological characteristics of study versus Z0011 population.

Parameters	Z0011 study intention to treat (*n* = 856)	Present study (*n* = 128)	Χ^2^	*p*
Age, years				
≤50, no. (%)	295 (35.2)	62 (48.4)	7.84	0.005
>50, no. (%)	544 (64.8)	66 (51.6)		
Clinical T stage, no. (%)				
T1	587 (69.3)	22 (17.2)	126.8	<0.001
T2	260 (30.7)	106 (82.8)		
Estrogen receptor, no. (%)				
ER+	659 (83)	108 (84.4)	0.14	0.69, *ns*
ER−	135 (17)	20 (15.6)		
Progesterone receptor, no. (%)				
PR+	534 (68.8)	105 (82)	9.2	0.003
PR−	242 (31.2)	23 (18)		
Modified Bloom-Richardson score, no (%)				
Grade 1	152 (23.8)	9 (7)	31.7	<0.001
Grade 2	306 (47.9)	54 (42.2)		
Grade 3	181 (28.3)	65 (50.8)		
Tumor type, no. (%)				
Infiltrating ductal	700 (80.3)	120 (93.8)	9.7	<0.001
Infiltrating lobular	63 (7.5)	5 (3.9)		
Others	77 (9.2)	3 (2.3)		
Size of SLN mets, no. (%)				
Micrometastasis	301 (41.2)	22 (17.2)	25.7	<0.001
Macrometastasis	430 (58.8)	106 (82.8)		
**Parameters**	**Z0011 ALND arm, intention to treat (*n* = 420)**	**Present study (*n* = 128)**	***X* ^2^**	***p***
Completion ALND, no. (%)				
Non SLN positive	97 (27)	62 (48.4)	18.04	<0.001
Non SLN negative	258 (73)	66 (51.6)		

ER = Estrogen receptor, PR = Progesterone receptor, ALND = Axillary lymph node dissection, SLN = Sentinel lymph node

**Table 2.** Clinicopathological characteristics of Group 1 (node negative ALND) versus Group 2 (node positive ALND).

Parameters	Group 1 (node negative ALND) (*n* = 66)	Group 2 (node positive ALND) (*n* = 62)	Χ^2^	*p* (univariate)	*p* (multivariate)
Age, years					
≤50, no. (%)	32 (48.5)	30 (48.4)	0.00	1.00, *ns*	0.77, *ns*
>50, no. (%)	34 (51.5)	32 (51.6)			
Clinical T stage,no. (%)					
T1	17 (25.8)	5 (8.1)	7.03	0.01	0.02
T2	49 (74.2)	57 (91.9)			
Estrogen receptor, no. (%)					
ER+	54 (81.8)	54 (87.1)	0.67	0.47, *ns*	0.16, *ns*
ER−	12 (18.2)	8 (12.9)			
Progesterone receptor, no. (%)					
PR+	51 (77.3)	54 (87.1)	2.09	0.17, *ns*	0.17, *ns*
PR−	15 (22.7)	8 (12.9)			
Modified Bloom-Richardson score, no (%)					
Grade 1	2 (3)	7 (11.3)		0.18 (Fisher exact test), *ns*	0.15, *ns*
Grade 2	30 (45.5)	24 (38.7)			
Grade 3	34 (51.5)	31 (50)			
Tumor type, no. (%)					
Infiltrating ductal	63 (95.5)	57 (91.9)		0.39 (Fisher exact test), *ns*	0.26, *ns*
Infiltrating lobular	1 (1.5)	4 (6.5)			
Others	2 (3)	1 (1.6)			
Size of SLN mets, no. (%)					
Micrometastasis	20 (30.3)	2 (3.2)	16.46	<0.001	0.001
Macrometastasis	46 (69.7)	60 (96.8)			

ER = Estrogen receptor, PR = Progesterone receptor, ALND = Axillary lymph node dissection, SLN = Sentinel lymph node

**Table 3.** Stage and adjuvant treatment decision between SLNB (only) versus SLNB + ALND.

Z0011 eligible patients, *n* = 128			
Parameters	SLNB only	SLNB + ALND	Remarks
Nodal stage, *n* (%)			
N1	128 (100)	103 (80.5)	Nodal stage changed in 19.5%.
N2	0	24 (18.8)	
N3	0	1 (0.8)	
TNM stage (AJCC 7th), *n* (%)			
Stage 2	128 (100)	103 (80.5)	TNM stage changed in 19.5%.
Stage 3	0	25 (19.5)	
MDT decision (chemotherapy), *n* (%)			
Yes	115 (89.8)	119 (93)	In 4/128 (3%), ALND changed the decision of chemotherapy.
No	13 (10.2)	9 (7)	
**Z0011 eligible + mastectomy, *n* = 176**			
Nodal stage, *n* (%)			
N1	176 (100)	143 (81.3)	Nodal stage changed in 18.7%.
N2	0	28 (15.9)	
N3	0	5 (2.8)	
TNM stage (AJCC 7th), *n* (%)			
Stage 2	176 (100)	143 (81.3)	TNM stage changed in 18.7%.
Stage 3	0	33 (18.7)	
MDT decision (chemotherapy), *n* (%)			
Yes	157 (89.8)	161 (91.5)	In 4/176 (2.2%), ALND changed the decision of chemotherapy.
No	19 (10.2)	15 (8.5)	
MDT decision (Radiotherapy), *n* = 48, *n* (%)			
Yes	43 (89.6)	43 (89.6)	No change
No	5 (10.4)	5 (10.4)	

SLNB = Sentinel lymph node biopsy, ALND = Axillary lymph node biopsy, MDT = Multi-disciplinary team
